# Transcriptome profiles of stem-like cells from primary breast cancers allow identification of ITGA7 as a predictive marker of chemotherapy response

**DOI:** 10.1038/s41416-021-01484-w

**Published:** 2021-07-12

**Authors:** Noha Gwili, Stacey J. Jones, Waleed Al Amri, Ian M. Carr, Sarah Harris, Brian V. Hogan, William E. Hughes, Baek Kim, Fiona E. Langlands, Rebecca A. Millican-Slater, Arindam Pramanik, James L. Thorne, Eldo T. Verghese, Geoff Wells, Mervat Hamza, Layla Younis, Nevine M. F. El Deeb, Thomas A. Hughes

**Affiliations:** 1 School of Medicine, University of Leeds, Leeds, UK; 2 Pathology Department, Faculty of Medicine, Alexandria University, Alexandria, Egypt; 3 Department of Breast Surgery, Leeds Teaching Hospitals NHS Trust, Leeds, UK; 4 Department of Histopathology and Cytopathology, The Royal Hospital, Muscat, Oman; 5 School of Physics and Astronomy, University of Leeds, Leeds, UK; 6 Children’s Medical Research Institute, Westmead, NSW Australia; 7 St. Vincent’s Clinical School, University of New South Wales, Sydney, Australia; 8 Department of Breast Surgery, Bradford Teaching Hospitals NHS Trust, Bradford, UK; 9 Department of Histopathology, St. James’s University Hospital, Leeds, UK; 10 School of Food Science and Nutrition, University of Leeds, Leeds, UK; 11 School of Pharmacy, University College London, London, UK

**Keywords:** Cancer stem cells, Chemotherapy, Breast cancer

## Abstract

**Background:**

Breast cancer stem cells (BCSCs) are drivers of therapy-resistance, therefore are responsible for poor survival. Molecular signatures of BCSCs from primary cancers remain undefined. Here, we identify the consistent transcriptome of primary BCSCs shared across breast cancer subtypes, and we examine the clinical relevance of ITGA7, one of the genes differentially expressed in BCSCs.

**Methods:**

Primary BCSCs were assessed using immunohistochemistry and fluorescently labelled using Aldefluor (*n* = 17). Transcriptomes of fluorescently sorted BCSCs and matched non-stem cancer cells were determined using RNA-seq (*n* = 6). ITGA7 expression was examined in breast cancers using immunohistochemistry (*n* = 305), and its functional role was tested using siRNA in breast cancer cells.

**Results:**

Proportions of BCSCs varied from 0 to 9.4%. 38 genes were significantly differentially expressed in BCSCs; genes were enriched for functions in vessel morphogenesis, motility, and metabolism. ITGA7 was found to be significantly downregulated in BCSCs, and low expression significantly correlated with reduced survival in patients treated with chemotherapy, and with chemoresistance in breast cancer cells in vitro.

**Conclusions:**

This study is the first to define the molecular profile of BCSCs from a range of primary breast cancers. ITGA7 acts as a predictive marker for chemotherapy response, in accordance with its downregulation in BCSCs.

## Background

Breast cancer is the most diagnosed cancer and leading cause of cancer death in women worldwide [1]. Primary cancers are typically treated with surgery mostly combined with systemic therapies, including cytotoxic chemotherapy in around one-third of cases, aiming to reduce distant recurrence risk [2]. Once metastases develop, these are terminal, although patients can survive for some years supported by a succession of further therapies [2].

Initiation, propagation and metastasis may be driven by rare cancer cells referred to as cancer stem, or stem-like, cells (CSCs), which have some properties associated with stem cells from healthy tissues, including self-renewal and multi-potent differentiation [3, 4]. The CSC model contends that CSCs are the key fully transformed and malignant cancer component that supports carcinogenesis through both self-renewal and limited differentiation into bulk tumour cells, which themselves do not have complete malignant properties [3, 5]. Importantly, CSCs are directly associated with metastases through their ability to seed new tumour foci to distant sites [5–7], in part by transitioning between epithelial and mesenchymal behaviours to invade and disseminate [8]. Also, CSCs are more resistant to radiotherapy and chemotherapy than bulk tumour cells and this is believed to be a key factor in recurrences [5, 9]. Therefore, improved knowledge concerning characteristics of breast cancer stem-like cells (BCSCs) will aid design of novel therapies targeting them, in order to reduce recurrences and improve outcomes [10].

BCSCs have been investigated in primary tumours, cell lines and mouse models using various markers, including CD44/CD24 [4, 6], CD133 [11], ITGA6 [12] and high expression [7] or activity [7, 13] of aldehyde dehydrogenase 1 (ALDH1) as assessed by Aldefluor assays. Interestingly, recent studies suggest that some markers identify different, although substantially overlapping BCSC populations. For example, CD44 high/CD24 low BCSCs are more mesenchymal-like, with lower proliferation rates and higher invasive capacities, while ALDH1 positive BCSCs are more epithelial-like, with higher proliferation and lower invasive capacities [8]. However, other data suggest that further BCSC subsets combine both mesenchymal and epithelial characteristics [14]. There is no consensus on the definitive markers for analysis of BCSCs, with different markers identifying groups of cells that are enriched in BCSC-properties but also demonstrate different behaviours [15]. Very few studies have characterised expression profiles from isolated BCSC and overwhelmingly only in cell lines [16–18], therefore molecular differences between BCSCs and bulk tumour cells in human breast cancers remain obscure. Only two published studies have defined transcriptomes of human primary BCSCs, involving either one patient [19], or two HER2-positive patients [20]. Here, we provide, transcriptome data for primary human BCSCs from multiple molecular and histopathological cancer subtypes, and we compare these to matched bulk tumour cells thereby defining key, subtype-independent, BCSC characteristics using a mixed cohort 3-times larger than the largest previous work [20]. We also demonstrate the utility of this profile, by examining impacts of one of the deregulated genes, ITGA7, in further cohorts and in tissue culture, thereby defining ITGA7 as a predictive marker of chemotherapy response, in accordance with the known chemoresistance of BCSCs [21].

## Methods

### Patients, ethics, clinical samples/data

Prospective work (ethical permission from Leeds [East] REC [15/YH/0025]): 17 female patients undergoing resections for primary breast cancer at St James’s University Hospital (Leeds) were recruited from 9/2016 to12/2016. Exclusion criteria were tumours estimated as <1 cm on pre-operative imaging, or neoadjuvant therapy. Two or three core biopsies were obtained from fresh cancer tissue immediately after excision. The 17 cases described are the entire cohort recruited for these experiments; we have not excluded any further cases for which assays failed. Cores were placed in RPMI (Thermo Fisher; Waltham, USA) (4 °C) and were processed immediately. Archival cancer blocks and clinicopathological data were collected from histopathology and hospital databases. Retrospective work (ethical permission from Leeds [East] REC [06/Q1206/180]): this cohort has been described previously [22]; brief details follow. Tissue microarrays (TMAs) were used containing treatment-naïve cancer tissue (three cores per case) from 305 patients treated with adjuvant chemotherapy, supported by clinicopathological data and outcomes follow up (Table S1). Disease-free survival (DFS) was defined as time from primary diagnosis to recurrence, while disease-specific survival was time from primary diagnosis to death from cancer. Patients gave informed, written consent for use of tissues/data in accordance with ethical permissions, and the study protocol conformed to the Declaration of Helsinki. Data are reported in accordance with REMARK where appropriate [23]. Figure S1 shows a flow-scheme clarifying cohorts used and which assays were performed.

### Aldefluor labelling and flow-cytometry/sorting

Single cell suspensions were prepared from core biopsies by mechanical and enzymatic digestion using GentleMACS dissociators and tumour dissociation kits (Miltenyi Biotech; Bergisch-Gladbach, Germany), according to the manufacturer’s instructions (further details in Supplementary methods), into total volumes of 1 ml. Total cell numbers ranged from 145,000 to >1.5 million. Stem-like cells were labelled based on ALDH activity by Aldefluor assays (StemCell technologies; Vancouver, Canada) [7, 13] according to the manufacturer’s instructions. Briefly, single cell suspensions were incubated with substrate BODIPY aminoacetaldehyde (BAAA), for 45 min (37 °C), both without (test) and with (control) 15 μM diethylaminobenzaldehyde (DEAB), a specific inhibitor of ALDH. Labelling of hemopoietic cells was achieved using 1/50 V450-labelled mouse anti-human CD45 (BD Biosciences, San Jose, USA), incubated for 30 min (4 °C), followed by washes. 10 μg/ml 7-Aminoactinomycin D (7-AAD) (LKT Labs, Saint Paul, USA) was added and incubated for 5 min (4 °C) in the dark immediately before analysis, in order to label lysed cells through nuclear staining. Cells were analysed or analysed/sorted immediately after completion of labelling using the Attune flow-cytometer (Applied Biosystems; Carlsbad, USA) or the Influx cell sorter (BD Biosciences; San Jose, USA). Analyses were of 10,000 to >850,000 events (greater when sorting), gating on live nucleate cells on forward scatter/side-scatter, live cells by excluding 7-AAD positives, and non-hemopoietic cells by excluding CD45 positives (Fig. S2). Aldefluor positive cells were defined using gates set for each individual sample, based on accepting ~1% positivity in matched DEAB-inhibited negative controls, with quoted values representing test minus control percentage. It should be noted that this gating strategy does not specifically exclude mesenchymal cells or normal breast epithelial cells. Sorted cells were stored as cell pellets at −70 °C before RNA extraction. Aldefluor fluorescence (BL1): excitation, 488 nm; emission, 530/30 nm LP filter. 7-AAD fluorescence (BL3): excitation, 488 nm; emission, 640 nm LP filter. CD45-V450 fluorescence (VL1): excitation, 405 nm; emission, 450/40 nm LP filter.

### Transcriptome profiling and analysis

RNA was extracted from BCSC (Aldefluor positive) or bulk cell (Aldefluor negative) populations and RNA-seq was performed and analysed as described in the Supplementary Methods. Sequencing data have been uploaded to NCBI BioProject, reference PRJNA642867.

### Immunohistochemistry

Immunohistochemistry was performed broadly as previously [24] and is described in the Supplementary Methods. In brief, sections were taken onto glass slides and were dewaxed (xylene) and rehydrated (descending ethanol grades). Antigens were retrieved by heating in citric buffer and slides were blocked in hydrogen peroxide. Slides were incubated with 1:50 mouse monoclonal anti-ALDH1 antibody (BD Biosciences; San Jose, USA) in Antibody Diluent (1 h room temperature) or 1:100 rabbit polyclonal anti-ITGA7 antibody (ab75224; Abcam; Cambridge, USA) in Antibody Diluent (overnight 4 °C). For ALDH1, IHC was completed using anti-mouse Envision reagents (Dako; Glostrup, Denmark) following the manufacturer’s protocols, while for ITGA7 SignalStain Boost IHC detection Reagent (HRP, Rabbit) and SignalStain DAB substrate were used (Cell Signalling Technology; Massachusetts, USA). Slides were counterstained with Mayer’s Haematoxylin (2 min). Finally, slides were washed, dehydrated (ascending grades of ethanol), cleared (xylene) and mounted in DPX (Fluka; Gillingham, UK). Sections were digitally scanned using ScanScopeXT (20×) and scored using Webscope (Aperio; Vista, USA). NG (specialist histopathologist) scored ALDH1. Cytoplasmic ALDH1 expression in tumour cells was assessed in terms of percentage of positively stained tumour cells and staining intensity, giving totals of 0–15. For ITGA7, SJJ and RAM-S (breast consultant histopathologist) scored and the scoring protocol was developed in consultation with RAM-S. Cytoplasmic and nuclear staining were scored separately, based on intensity and proportion, giving final scores of 0–8. SJJ scored all cores, while RAM-S scored 10% of cores independently; Cohen’s Kappa statistic indicated near perfect agreement between scorers (0.83 for nuclear; 0.88 for cytoplasmic ITGA7), demonstrating scoring was robust and reproducible. ITGA7 scores for each case were means of scores from each core representing that case.

### In silico analyses: expression data mining and structure visualisation

METABRIC data were accessed on 7/2/2021 via cbioportal [25], as reported previously [26]. Records with ITGA7 expression data and suitable clinical annotation were identified (*n* = 1903). Cases were dichotimised into low and high ITGA7 expression using receiver operator curve analyses [27]. Visualisation of molecular structures from X-ray crystallography and homology modelling was performed using the Chimera software [28].

### Tissue culture, transfections and MTT assays

MCF7 cells were purchased (ATCC) and cultured in DMEM, 10% FCS (Thermo Fisher; Waltham, USA), 95% air/5% CO_2_ at 37 °C. Cell line identity was confirmed (STR profiles, Leeds Genomics Service) and cultures were consistently Mycoplasma negative (MycoAlert; Lonza; Basel, Switzerland). Cells were transfected with ITGA7 specific siRNA (#SR320703) or non-targeted control siRNA from OriGene (Rockville, USA) using Lipofectamine 3000 in OptiMEM media (ThermoFisher, MA, USA) for 18 h, before medium was replaced with full fresh medium. Epirubicin hydrochloride (Sigma-Aldrich; St Louis, USA) was prepared as a 10 mM stock in water, and was diluted in medium for treatment of cells for up to 72 h. MTT (3-(4,5-dimethylthiazol-2-yl)−2,5-diphenyltetrazolium bromide) assays were performed as previously [22].

### Western blots and immunofluorescence

Cells were washed in PBS (4 °C) and then incubated with lysis buffer (10 mM HEPES pH 7.9, 10 mM KCl, 0.1 mM EDTA, 0.4% IGEPAL CA-630 [Sigma-Aldrich; St Louis, USA], 1 mM DTT and Halt protease/phosphatase inhibitor [ThermoFisher; Waltham, USA]) for 10 min (room temperature). Cells and buffer were collected and centrifuged at 15000 g for 3 min (4 °C). Proteins within supernatants were quantified using Bradford reagent (Merck; New Jersey, USA). Proteins were denatured in Laemmli buffer (ThermoFisher; Waltham, USA), 5 min at 90 °C, and equal masses in each lane were separated on 4–12% polyacrylamide gels (BioRad; Watford, UK). Proteins were transferred to PVDF and blocked with 5% non-fat milk in TBST (Tris Buffered Saline, 0.1% Tween-20) for 45 min. Membranes were incubated with antibody against ITGA7 (as above) or rabbit monoclonal antibody against β-actin (4970S; Cell Signalling Technologies; Beverly, USA) at 1:2000 in TBST overnight (4 °C), and then with HRP-tagged secondary antibody (Cell Signalling Technologies; Beverly, USA) at 1:4000 in TBST for 3 h (room temperature). Results were visualised using Pierce ECL reagents (ThermoFisher, Waltham, USA) by ChemiDoc (BioRad; Watford, UK). Densitometry was performed using ImageJ (NIH Freeware, USA). For immunofluorescence, cells were seeded on coverslips. Cells were washed in PBS and fixed with 4% paraformaldehyde (Merck; New Jersey, USA) in PBS for 10 min (room temperature). Cells were washed (PBS, x3) and permeablized with 0.2% Triton X-100 (Merck; New Jersey, USA) in PBS at 4 °C for 10 min. Cells were washed (PBS) and blocked with 5% FBS in PBS (4 °C, 1 h). Cells were incubated with antibody against ITGA7 (as above) at 1:200 dilution in wash buffer (0.5% FBS, 0.05% Tween-20 in PBS) overnight (4 °C). Further washes were performed (wash buffer) and cells were incubated with 4 µg/ml AlexaFluor-633 labelled goat anti-rabbit secondary antibody (A-21070; ThermoFisher; Waltham, USA) for 1 h (room temperature; dark). Cells were then washed (wash buffer), mounted in 50% glycerol containing 2 µg/ml DAPI, and analysed using confocal microscopy (Nikon A1R; Nikon; Melville, USA).

### Statistical analyses

Statistical analyses were performed using SPSS v19 (SPSS; Chicago, USA) or Prism (v7 or v8) (GraphPad, San Diego, USA). Statistical tests used are described in figure legends, in the results text, or in the Supplementary methods.

## Results

### Fluorescent labelling of stem-like cancer cells from primary breast cancers

Our first aim was to establish protocols by which BCSCs can be labelled in primary breast cancers to allow their separation from bulk tumour cells and from stromal (non-cancer) cells. To achieve this, we accessed fresh primary breast tumour tissue from an initial cohort of 11 cases. Tissues were treated immediately to form single cell suspensions, and stem-like cells were labelled using Aldefluor assays that fluorescently label BCSCs because of their strong ALDH activity [7, 13]. A key control is use of the specific ALDH inhibitor, DEAB. Cells were treated for Aldefluor labelling in parallel with and without DEAB, allowing confirmation that fluorescent-labelling was associated with ALDH activity by comparison with inhibited controls. Flow-cytometry was used to assess proportions of cells that were fluorescently labelled dependently on ALDH activity. For our first 3 cases, we gated initially on nucleated cells using forward and side scatter, thereby excluding red blood cells and cell debris, and then assessed proportions of Aldefluor positivity. For subsequent cases, we added further complexity, by additionally gating on cells that did not take up the DNA-binding dye 7-AAD, thereby excluding any non-viable cells that would be dye permeable, and gating on cells that were negative for CD45, thereby excluding hemopoietic cells (Fig. S2). Having refined this protocol, we then performed assays on 6 further cases, and used fluorescence-activated cell sorting to collect cells from both Aldefluor positive (BCSCs) and Aldefluor negative (bulk cancer cell) populations. Table 1 shows clinicopathological features of all 17 cases (the initial 11 cases [cases 1-11], and the subsequent 6 [cases 12–17] from which we sorted BCSCs), along with proportions of cells defined as Aldefluor positive (BCSCs). These proportions varied substantially from 0 to 9.4% (mean 3.8%) - values that are in line with studies using patient-derived xenografts [29, 30] that provide the best available comparator.

**Table 1:** Clinical features of the study cohort (*n* = 17) with proportion of tumour cells showing Aldefluor positivity, the gating strategy, and the ALDH1 immuno-score.

	Histology	Grade	LN status	ER status	HER2 status	Subtype	Ald+ %	Gating	ALDH1 score
1	Mucinous	1	Neg	Pos	Neg	Luminal	7.62	ALDEFLUOR	0
2	Invasive ductal, NST	2	Pos	Pos	Neg	Luminal	4.68	ALDEFLUOR	1
3	Invasive lob., pleomorphic	2	Neg	Pos	Neg	Luminal	5.5	ALDEFLUOR	4
4	Invasive lob., classic	2	Neg	Pos	Neg	Luminal	3.58	7-AAD, ALDEFLUOR	0
5	Invasive ductal, NST	1	Neg	Pos	Neg	Luminal	0.07	7-AAD, CD45, ALDEFLUOR	2
6	Invasive ductal, NST	2	Pos	Pos	Neg	Luminal	4.83	7-AAD, CD45, ALDEFLUOR	1
7	Invasive ductal, NST	3	Pos	Neg	Neg	Triple negative	0	7-AAD, CD45, ALDEFLUOR	12
8	Encapsulated papillary	2	Neg	Pos	Neg	Luminal	3.36	7-AAD, CD45, ALDEFLUOR	1
9	Invasive lob., classic	2	Pos	Pos	Neg	Luminal	2.31	7-AAD, CD45, ALDEFLUOR	0
10	Invasive ductal, NST	2	Neg	Pos	Neg	Luminal	0.54	7-AAD, CD45, ALDEFLUOR	4
11	Invasive ductal, NST	2	Pos	Pos	Neg	Luminal	3.57	7-AAD, CD45, ALDEFLUOR	1
12	Invasive ductal, NST	2	Pos	Pos	Pos	Luminal B (HER2+)	4.49	7-AAD, CD45, ALDEFLUOR	4
13	Invasive ductal, NST	3	Pos	Pos	Pos	Luminal B (HER2 + )	8.24	7-AAD, CD45, ALDEFLUOR	6
14	Invasive lob., pleomorphic	2	Neg	Pos	Neg	Luminal	2.5	7-AAD, CD45, ALDEFLUOR	1
15	Invasive lob., classic	2	Pos	Pos	Neg	Luminal	0.5	7-AAD, CD45, ALDEFLUOR	0
16	Solid papillary	2	Neg	Pos	Neg	Luminal	3.37	7-AAD, CD45, ALDEFLUOR	2
17	Invasive ductal, NST	2	Neg	Neg	Neg	Triple negative	9.38	7-AAD, CD45, ALDEFLUOR	4

*LN* lymph node, *Ald+ %* % of cells defined as Aldefluor positive, *ALDH1 score* ALDH1 expression score defined by immunohistochemistry, *NST* no special type, *lob.* lobular, *Pos* positive, *Neg* negative.

### ALDH1 protein expression did not correlate with ALDH activity

Next, we were interested to establish whether our determination of BCSC populations was a simple reflection of ALDH1 expression, or whether the activity assay gives an additionally subtle assessment of true functional relevance. Therefore, we assessed ALDH1 expression in tumour tissues from our 17 cases using immunohistochemistry and compared this with Aldefluor positivity. ALDH1 positive staining was detected in tumour cell cytoplasm and was quantified in terms of percentage of positively stained tumour cells and their intensity, which were combined to give scores from 0 (no staining) to 15 (strong staining in >66% of cells), as previously for ALDH1 [7, 13]. Some ALDH1 expression was also seen focally in adjacent normal tissue and in stromal cells within the cancers, although this was not quantified. Representative ALDH1 staining is shown (Fig. S3).

ALDH1 expression in tumour cells was observed in 13 cases (76.5%) and was detectable in a minority of cells in all but one of these. The median percentage positivity was 1% (range 0–65%), which is compatible with the literature [13] and the concept that stem-like cells are rare. Intensity of positive tumour cell staining varied from weak (6 cases), moderate (6 cases), to strong (1 case). Overall scores, therefore, ranged from 0 to 12 (median 1) (Table 1). Correlations between ALDH activity, assessed as Aldefluor positive proportion, and ALDH1 expression, assessed by IHC as either combined proportion/intensity scores or—more simply—percentages of ALDH1 positive tumour cells, were determined using Spearman’s correlation tests. No significant correlations were found using either measure of ALDH1 expression (proportion/intensity scores *r* = 0.08; *p* = 0.75; percentage positivity *r* = 0.12, *p* = 0.64). We concluded that assessment of proportions of Aldefluor positivity does not simply reflect total ALDH1 protein expression but provides a more subtle functional assay, as has been reported previously [31, 32].

### Stem-like and bulk breast cancer cells differ significantly in their transcriptomes

**Fig. 1:**
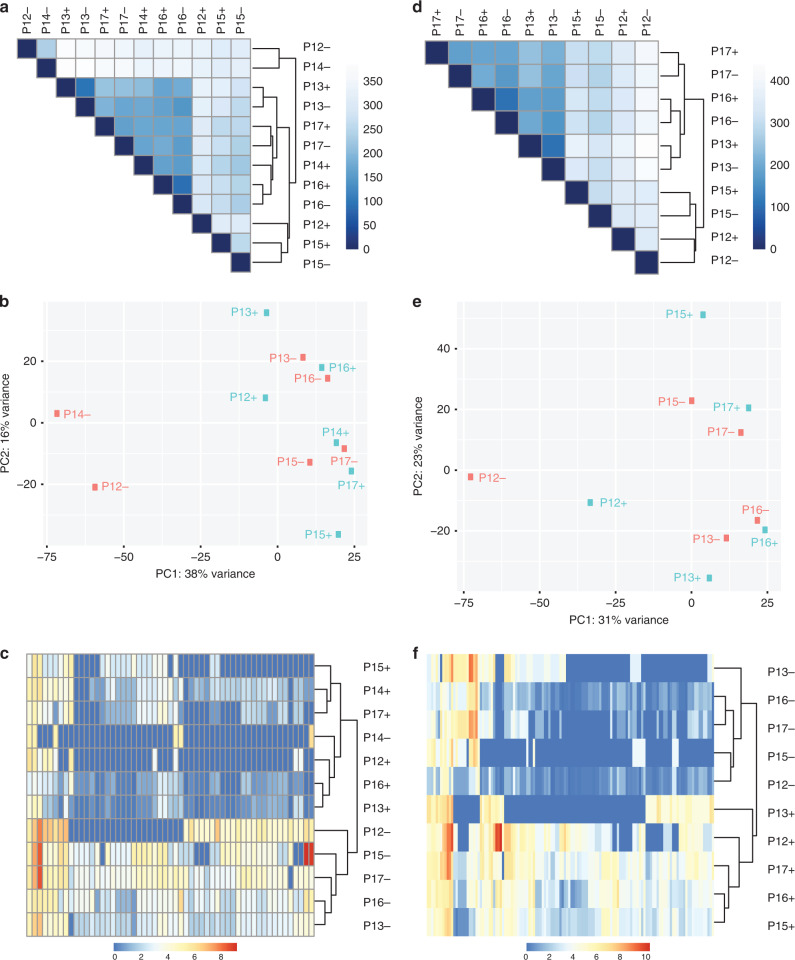
Expression profiles define consistent features of BCSCs. BCSCs (Aldefluor positive, +) and matched bulk (Aldefluor negative, −) cancer cells were sorted by FACS from 6 cancers (patients, P, 12 through to 17). Expression profiles were determined by RNA-seq. Analyses were performed on all 6 pairs of samples (**a**–**c**) or only 5 pairs, excluding P14 (**d**–**f**). **a**, **d** Unsupervised hierarchical clustering was performed to investigate the relationships between the samples. Dendrograms and heat maps are shown. **b**, **e** Principal component analysis (PCA) was performed to investigate the relationships between the samples. **c**, **f** Supervised hierarchical clustering was performed using the genes significantly differentially expressed between paired BCSCs and bulk samples. Dendrograms and heat maps are shown.

Our next aim was to define transcriptomes of stem-like and, for comparison, matched bulk cancer cells. Therefore, we extracted total RNA from BCSC (Aldefluor positive) and the bulk cancer cell (Aldefluor negative) populations sorted from the final 6 breast cancers of our cohort. These cases represented a variety of histologies and examples of ER positive and negative cancers, and HER2 positive and negative cancers (Table 1); although ER-negative/HER2-positive disease was not included. RNA was sequenced and expression profiles for each sample were determined. The numbers of aligned sequencing reads for each sample are shown in Table S2. Relationships between these profiles were initially analysed using unsupervised hierarchical clustering (Fig. 1a) and principal component analyses (PCA) (Fig. 1b). These analyses revealed that overall, pairs of matched samples from individual cases were more closely related to each other, than relationships within the BCSC or bulk compartments across cases, as evidenced by paired hierarchical clustering in 4 cases, and 3 of the Aldefluor positive samples being closest to their paired sample in PCA. However, 2 cases showed little evidence of pairing, with patient 14 demonstrating particularly extreme PCA separation of matched samples. Unsupervised clustering and PCA were repeated excluding this case (Fig. 1d, e); matched BCSC and non-stem profiles now paired perfectly for the remaining cases in both clustering and PCA. We also used PCA to test whether BCSC receptor status was a key factor in defining their characteristics; PCA was repeated with only the 6 BCSCs samples and groupings of HER2-positive vs HER2-negative, and ER-positive vs ER-negative samples were examined (Fig. S4). There was some suggestion of the samples grouping according to HER2 status, although the trend was weak with substantial variation within the groups. By contrast, there was no suggestion of separation by ER status, although this analysis is compromised by the fact that there was only one ER-negative case.

Next, we analysed transcriptomes to identify significantly differentially expressed transcripts between BCSCs and bulk tumour cells, using all 6 paired samples, or only 5 pairs (excluding case 14). After correction for multiple testing, 55 differentially expressed transcripts were identified using all samples (54 downregulated in BCSCs, and—surprisingly—only 1 upregulated) and 130 transcripts were identified using the 5 pairs (118 downregulated, 12 upregulated). Transcripts are listed in Table S3, along with mean fold-differences in expression and statistical significances (multiple testing adjusted *p* values). 95% of transcripts from analysis of all samples were also present on the 5 pairs list, while the 5 pairs list was 59% unique. Supervised hierarchical clustering was performed using these differentially expressed genes (Fig. 1c, f). Using data from all samples (Fig. 1c), expression of these transcripts clustered all BCSC samples together, although this cluster also included the bulk cell sample from case 14, again demonstrating that this case was an outlier. When case 14 was excluded (Fig. 1f), BCSC and bulk samples clustered separately accurately. It is tempting to speculate as to why case 14 appears to behave differently; this might relate to it being the only representative of lobular pleomorphic histology within the sequencing dataset, however, the analysis is underpowered to secure this conclusion.

The 8 most up- and downregulated genes within BCSCs from both analyses are listed (Table 2), when available, which included both coding and non-coding genes showing up to 1000-fold differential expression. Upregulated genes, although fewer than downregulated, included PDGFRA, which has previously been reported as upregulated in BCSCs [19, 33], and SFRP2, which can promote stem-like behaviours such as induction and survival of breast metastases [34]. Whereas, downregulated genes included GJA4, which is a component of gap junctions that are downregulated in CSCs [35], and BTNL9 and ITGA7, which are proposed tumour suppressors in breast [36, 37]. It is interesting to note that ALDH transcripts were not significantly differentially expressed after correction for multiple testing, although expression of both ALDH1A1 and ALDH1A3, ALDH family members thought to contribute most to stem phenotypes [38], were significantly upregulated in BCSCs before correction (means of 5.8 and 3.4-fold respectively with all cases, and means of 9.8 and 6.5-fold with 5 cases). We concluded that we had successfully identified transcripts associated with BCSCs from a range of primary breast cancers.

**Table 2:** The most up- or downregulated genes in BCSCs compared to matched bulk cancer cells.

	Downregulated		Upregulated	
	Gene	Mean fold change	Gene	Mean fold change
All	HBA2	207	LINC01279	5.3
	HBA1	175		
	GJA4	57		
	NDUFA4L2	42		
	BTNL9	39		
	ANGPT2	25		
	ITGA7	24		
	ROBO4	22		
5 pairs	HBB	1053	PDGFRA	8.8
	HBA2	303	DCN	8.8
	HBA1	183	LUM	7.8
	GJA4	83	SFRP2	6.3
	RGS5	66	LINC01279	5.9
	CDH6	48	RARRES2	4.0
	ABCB1	46	GFPT2	3.9
	BTNL9	40	COX8A	2.2

Expression in BCSCs and matched bulk cancer cells was compared in all 6 cases (All) or in only 5 cases (5 pairs). The 8 most up- or downregulated genes are listed (when 8 were available), along with mean fold-changes.

### Genes associated with BCSCs are enriched for specific functions

Next, we were interested to examine whether specific molecular functions were over-represented within the differentially expressed transcripts, which would give insight into how BCSCs functionally differ from bulk cancer cells. Differentially expressed transcripts were resolved into differentially expressed genes, noting that many were alternative-splices from fewer genes. 38 and 88 separate genes were included within the differentially expressed transcripts from all 6 cases or the 5 cases respectively. The lists were highly overlapping, with only 2 genes on the shorter list not represented on the longer. Differentially expressed genes were analysed for significant over-representation of specific gene ontology annotations, when compared to the pooled transcriptome of all samples. There were 25 and 131 significantly over-represented terms for genes from 6 or 5 cases respectively (Table S4). Many of these ontologies can be described in three broad categories: developmental regulation of vessels (including the 3 most significantly over-represented ontologies on both lists: cardiovascular system development, blood vessel development, tube morphogenesis); cell motility and migration (including regulation of cell motility, regulation of [epithelial/endothelial] cell migration, regulation of locomotion) and metabolism (including regulation of phospholipid metabolic process, hydrogen peroxide catabolic process, cellular oxidant detoxification). A further significantly over-represented ontology worth highlighting was oxygen transport, since this contained all three of the most highly differentially expressed genes (HBB, HBA1, HBA2; Table 2). We concluded that BCSCs show deregulation of a wide-range of cellular processes.

### ITGA7 is downregulated in stem cells and is implicated as a mediator of chemoresponse

From the genes differentially expressed between BCSC and non-stem compartments, we were particularly interested in potential prognostic and therapy predictive impacts of ITGA7, since it was previously reported as a tumour suppressor in breast cancer [37], and we had also identified ITGA7 somatic mutations, namely L36V and R157Q, that showed chemotherapy-induced selection in breast cancer [39]. We now aimed to examine in detail potential implications on protein function of these mutations.

ITGA7 is thought to function as a heterodimer with ITGB1 but its structure has not been solved; however, structures for the related ITGAV/ITGB3 heterodimer are available [40, 41], as are homology models of ITGA7 (swiss model ID Q13683 using the homology model based on the 3fcs.2.A template residues 34-1089) and ITGB3 (swiss model ID P05556 using the homology model based on the 4g1m.1.B template residues 25–727). To construct a model of the ITGA7/ITGB1 complex, we overlaid the ITGA7 A-chain of the homology model with the A-chain of the αvβ3 crystal structure (pdb ID 3IJE). Likewise, the ITGB1 homology model was superimposed on the B-chain of the same crystal structure. We mapped the ITGA7 somatic mutations onto this structure, noting that both variants occur in regions that are highly conserved. Both are located at key molecular recognition interfaces (Fig. S5A, Video S1), and so are well positioned structurally to influence the stability of the ITG alpha chain and its interaction with other substrates. The residue equivalent to L36 is located at the binding interface between the N-terminal end of the β-propeller and the ‘thigh’ domain [41] of the ITG alpha chain. The bulky leucine sidechain occupies a hydrophobic cavity adjacent to the domain interface (Fig. S5B) that would be only partially occupied by the more compact valine in the L36V variant. Moreover, the positively charged R157 in the β-propeller domain in the alpha chain is located at the binding interface with the ‘βA’ domain [41] of ITGB1, and is stabilised by charge-charge interactions with the negatively charged E120 and E145 in the A-chain, which would be absent in the R157Q variant (Fig. S5C, D). We concluded that these mutations have likely functional impact on ITGA7, and therefore that ITGA7 activity is a potential regulator of breast cancer chemoresponse, based on therapy-induced selection of these mutations [39]; this is compatible with differential function in the stem compartment since CSCs are known to be chemoresistant [9, 21].

**Fig. 2:**
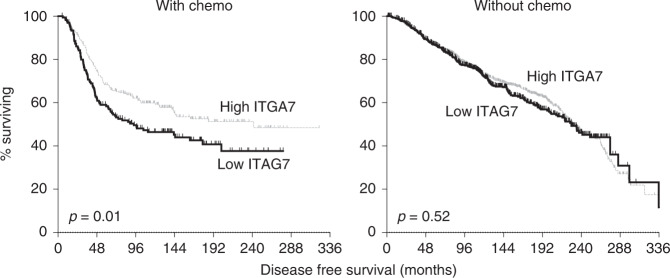
ITGA7 expression predicted disease-free survival in breast cancer after chemotherapy, but not after other treatments. METABRIC transcriptomic data for breast cancers were accessed via cbioportal and records with ITGA7 expression data and suitable clinical annotation were identified (*n* = 1903). Cases were split into those treated with chemotherapy (left plot; *n* = 396) and those treated without (right plot; *n* = 1507), and were dichotimised into low and high ITGA7 expression using receiver operator curve analyses. Kaplan–Meier survival analyses were performed and significance was assessed using Log-Rank Mantel-Cox tests.

Next, we assessed whether ITGA7 expression impacted on cancer outcomes using publicly-available transcriptome data for primary breast cancer samples. Using the METABRIC dataset, we tested whether ITGA7 expression levels correlated with disease-free survival in a cohort of breast cancer cases (*n* = 1903; Fig. S6), or in the same cases separating them into those annotated as receiving chemotherapy (*n* = 396) and those not so annotated (*n* = 1507) (Fig. 2). We found that ITGA7 expression did not impact significantly on survival in the total cohort (Fig. S6), but that low expression significantly correlated with reduced survival in patients treated with chemotherapy (*p* = 0.01; Fig. 2), but not in those who were not so treated, potentially indicative of specific roles in chemoresistance. We also examined the ER-positive and ER-negative chemotherapy-treated patients separately (Fig. S7); the correlation with survival was evident only in the larger ER-negative group. We concluded that ITGA7 was a strong candidate mediator of chemotherapy response in breast cancer, and therefore worthy of direct experimental testing.

### ITGA7 expression in cancer cells correlates with disease-free survival after chemotherapy

**Fig. 3:**
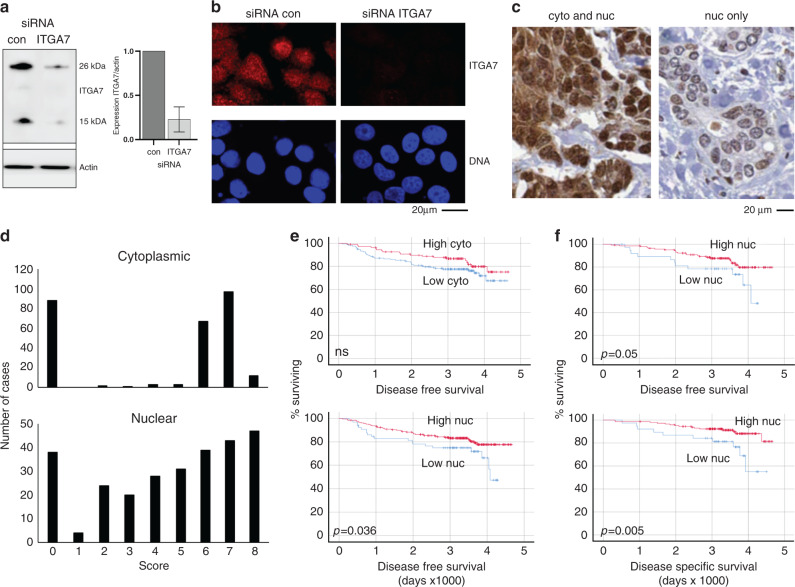
Low nuclear ITGA7 protein expression, especially in ER-positive disease, correlated with poor survival after chemotherapy. **a**, **b** MCF7 cells were transfected with control siRNA or siRNA targeted against ITGA7. ITGA7 expression was analysed using western blots (**a**) or immunofluorescence (**b**) using actin or the DNA stain DAPI respectively as a counter-stain. Relative ITGA7 expression after transfection with either control or ITGA7-targeted siRNA was quantified by densitometry from three independent experiments (**a**, right panel). **c**–**f** Breast tumour resection tissue from 305 breast cancer patients subsequently treated with adjuvant chemotherapy were stained for ITGA7 expression using immunohistochemistry (brown). Tissue was counterstained with Mayer’s Haematoxylin (blue). ITGA7 expression in cytoplasm and nucleus were scored separately on a scale of 0–8. **c** Representative staining is shown: left image scored cytoplasmic 7, nuclear 8; right image scored cytoplasmic 0, nuclear 5. **d** Distributions of scores in the cytoplasm (top) and nucleus (bottom) are shown. **e**, **f** Cases were dichotomised into two groups based on low or high expression using cut offs defined by receiver operator curve analyses. Kaplan–Meier survival analyses were performed to determine whether expression of ITGA7 in either cytoplasm (cyto) or nucleus (nuc) was significantly related to disease-free or disease-specific survival as labelled using either the whole cohort (*n* = 305; **e**) or the ER-positive cases only (*n* = 207; **f**). *p* values were determined using log rank tests; ns denotes not significant.

Our next aim was to test in a further cohort of breast cancers whether levels of ITGA7 protein expression were differentially associated with histopathological features of tumours, or with cancer outcomes specifically after chemotherapy. First, we tested the specificity of an ITGA7 antibody [42], with a view to using this in immunohistochemical analyses. We transfected the breast cancer cell line MCF7 with siRNAs targeting ITGA7, or with control non-targeting siRNAs, and assessed ITGA7 expression using this antibody by Western blot and immunofluorescence (Fig. 3a, b). The antibody detected a main ITGA7 species of ~26 kDa, which is the predicted size of the C–terminal portion of the protein resulting from a well-characterised proteolytic cleavage [43]; the epitope for this antibody is contained in this C-terminal end. In addition, a smaller fragment was detected. Both bands were specific to ITGA7 as indicated by their reduced expression after targeted knock-down. By immunofluorescence, ITGA7 was detected in the cytoplasm, and—surprisingly—the nuclei of cells. Critically, expression in both compartments was shown to be specific to ITGA7, as expression of both was dramatically reduced after targeted knock-down (Fig. 3b). We concluded that the antibody is specific for ITGA7 and therefore suitable for use in immunohistochemistry, and that expression in both cytoplasmic and nuclear compartments may be of interest. Nuclear functions have previously been reported for various integrins [44–46].

Next, we examined expression in 305 breast cancers, encompassing a variety of histopathological features, that were all treated with cytotoxic chemotherapy sometimes combined with other treatments according to individual tumour subtypes. Clinical and pathological features of this cohort have been published previously [22], and are summarised in Table S1. Tumour tissue samples were collected into tissue microarrays, with three separate tissue cores representing each case. Expression of ITGA7 was examined using immunohistochemistry; staining was cytoplasmic, with some accentuated staining at the membrane, while nuclear staining was also detected (Fig. 3c). Staining was scored taking into account proportions of tumour cells staining positively, and their intensity using the Allred system, assessing cytoplasmic and nuclear compartments separately. Distributions of scores are shown in Fig. 3d, demonstrating a near binary distribution in the cytoplasm, tending to be either absent or with medium or strong expression in the majority of cells, while by contrast nuclear expression was more evenly distributed across the scores. Despite these different distributions, cytoplasmic and nuclear expression were significantly correlated (Spearman’s coefficient 0.66, *p* = 0.01). ITGA7 expression was also tested for correlations with the standard prognostic factors tumour grade, lymph node status, and oestrogen-receptor status (Table S5). The only significant finding was nuclear ITGA7 demonstrated an extremely weak, and only just significant, negative correlation with tumour grade (Spearman’s coefficient −0.12, *p* = 0.04), although significance was lost after correction for multiple testing. Overall, we concluded that ITGA7 levels were independent of these factors.

Kaplan–Meier survival analyses were performed to determine whether expression of ITGA7 was significantly related to survival. Cut-offs were applied to dichotomise patients into groups with low or high expression. These cut-offs were defined objectively using receiver operator curve analyses to give the best balance between sensitivity and specificity for prediction of clinical outcome [27] (see Table S6 for cut off values). Relatively low ITGA7 expression in nuclei was significantly associated with shorter disease-free survival, by a mean of 647 days (*p* = 0.036; Fig. 3e bottom panel). This trend was also visible for cytoplasmic expression, although this was not significant, and the difference in survival was less (341 days; Fig. 3e top panel). For disease-specific survival, neither nuclear or cytoplasmic ITGA7 were significantly associated with outcome, although the same trend for low expression being associated with shorter survival was visible (*p* = 0.063 and 0.065 respectively; Fig. S8). Interestingly, when analyses were limited to ER-positive cases only (*n* = 207), nuclear ITGA7 expression was significantly associated with both disease free (Fig. 3f top panel, *p* = 0.05) and disease-specific survival (Fig. 3f bottom panel, *p* = 0.005). By contrast, ITGA7 was unrelated to either measure of outcome in the smaller ER-negative group (*n* = 98; Table S7). Surprisingly, this dependence on ER status is the opposite to our findings in the METABRIC dataset, where we found the correlation in the ER-negative group only. It should be noted that analysis of the METABRIC cohort used transcript levels from whole tissue samples, while for our cohort we have assessed nuclear protein levels in the cancer cells only, therefore some differences may be expected. We concluded that levels of ITGA7 protein, specifically within the nucleus and especially in oestrogen-receptor positive cases, were associated with survival after chemotherapy.

### Knock-down of ITGA7 increases chemotherapy resistance in breast cancer cell lines

**Fig. 4:**
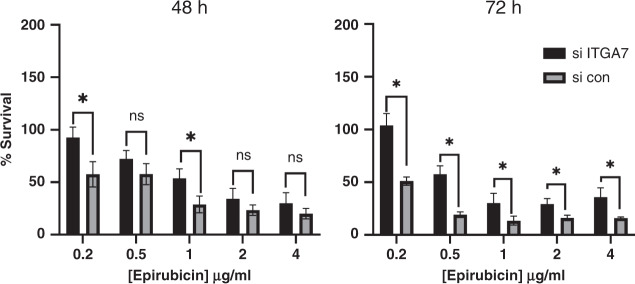
Reduced ITGA7 expression protects breast cancer cells from chemotherapy. MCF7 cells were transfected with siRNA targeted against ITGA7 (si ITGA7) or with nontargeting siRNA control (con). Cells were treated with doses of epirubicin 24 h after transfection, for a further 48 h (left) or 72 h (right) before relative cell survival was determined using MTT assays. Error bars represent SEM of three fully independent experiments. * Indicates significant differences at specific doses (*p* < 0.05; Mann–Whitney test). ns denotes not significant.

Next, we aimed to test directly whether expression levels of ITGA7 are associated with differential sensitivity of breast cancer cells to cytotoxic chemotherapy. We used the oestrogen-receptor positive cell line, MCF7, based on our observation that the association of ITGA7 with survival after chemotherapy was strongest in oestrogen-receptor positive cancers, and we used the anthracycline epirubicin as a representative chemotherapy agent, since this class of agents is used in the vast majority of breast cancer cases that receive chemotherapy. Cells were transfected with siRNAs targeting ITGA7 or with control non-targeting siRNAs, and sensitivity to a range of doses of epirubicin was determined using MTT assays after 48 or 72 h (Fig. 4). Efficacy of this targeted knock-down has already been demonstrated (Fig. 3a, b). Knock-down of ITGA7 was associated with significant protection of cells from the toxic effects of epirubicin at two doses after 48 h (although the trend is maintained with all doses), and at all doses after 72 h (*p* < 0.05). We concluded that reduced ITGA7 was associated with cancer cell resistance to epirubicin, which is compatible with our clinical data demonstrating low ITGA7 expression was associated with poor outcomes after chemotherapy (Fig. 3e, f).

## Discussion

We present the first published analyses of transcriptomes from BCSC isolated from a range of primary breast cancer subtypes. The cancer cases we examined (Table 1) included invasive ductal carcinomas of no special type, the commonest breast cancer histopathological classification [47], as well as rarer types (lobular and papillary carcinomas). We also included cases that were positive or negative for ER or HER2 expression, the key markers used to stratify to different therapies [2]. Therefore, our cohort covers much of the diversity that is characteristic of breast cancer; yet despite this, we find consistent BCSC features. Only two previous studies have examined BCSC transcriptomes from primary breast cancers [19, 20]. One study investigated CD44 high/CD24 low BCSCs from one ER-positive case [19]. Despite the difference in CSC marker and the diversity of our cohort, there were findings in common with our work; PDGFRA was upregulated, and JAG2 downregulated (Table S3) in BCSCs in both studies and similar deregulated pathways were identified, such as tissue morphogenesis and regulation of cell migration (Table S4). The PDGF pathway is already an established cancer therapeutic target, although this has not been linked specifically to BCSCs, and breast clinical trials are underway [48]. The other previous study of BCSC transcriptomes also used the markers CD44 high/CD24 low in two HER2-positive/ER-negative cancers [20]. There were few similar findings to our work, although expression of EMCN and MMRN2 were downregulated in BCSCs in both studies; this relative lack of commonality may reflect our lack of HER2-positive/ER-negative cases. It is also important to recognise that differences in markers used to identify BCSCs may be a critical source of lack of consistency between studies, with CD44 high/CD24 low cells known to differ from, although overlap with Aldefluor positive cells [15]. Of particular interest are reports that these two key types of BCSCs differ in cancer tissue distribution, with CD44 high/CD24 low cells being more prevalent at invasive edges while Aldefluor positive cells reside in the interior [8], and differ in relative prevalence across breast cancer molecular subtypes [49]. In this context, it may be important to interpret our results initially in the context of Aldefluor positive BCSCs specifically, and relating to the molecular subtypes we include, although we do demonstrate wider applicability through our follow up work on ITGA7.

Unexpectedly, we found that genes expressing haemoglobin chains (HBB, HBA1, HBA2) were the most differentially-expressed genes, each showing more than 100-fold downregulation in BCSCs. We considered whether this could have been caused by contamination of non-stem compartments with hematopoietic cells, despite experimental protocols designed to eliminate this by positive selection on live nucleate cells and negative selection on the hematopoietic marker CD45. In fact, our finding is supported by publications on expression of each of these genes in epithelial cancer cells, including from cervix, prostate, lung, and breast, and a growing hypothesis that this represents a mechanism for reducing oxidative stress-induced cellular damage [50–54]. This work is most advanced in breast cancer for HBB, although reports are conflicting; HBB has been shown to exhibit some characteristics of a tumour suppressor [55, 56], while others have shown expression to correlate positively with aggressive cancer behaviours such as proliferation [52, 53]. Our data may resolve these conflicts, as we find relatively high expression in bulk cancer cells, perhaps involved with proliferation, but greatly reduced expression in primary stem compartments that are relatively quiescent.

Other examples where overall tissue expression may have previously obscured potential roles within BCSCs are DCN and LUM, which encode the proteoglycans decorin and lumican. We find these to be among the most substantially upregulated genes in BCSCs (Table 2). This is in accordance with published data for both proteins in stem compartments of glioblastoma and neuroblastoma [57], and for DCN in colon cancer [58]. In addition, high DCN expression has been associated with stem-like characteristics of chemoresistance and invasion in oral [59] and bladder cancers [60]. By contrast, in breast cancer, high expression of either protein in tumour tissue, assessed by Western blots, was associated with good outcomes [61], and adenoviral overexpression of DCN [62] or treatment with recombinant DCN [63] have even been tested pre-clinically as therapies. However, both proteins are expressed highly in stromal cells and matrix, and these are the likely source of correlations with outcome [64] and the main target of exogenous protein [63]. When DCN expression specifically in cancer cells was assessed, high DCN correlated significantly with reduced survival [65], which is compatible with our observed high expression in BCSCs.

We focused further on ITGA7 since it was significantly and consistently downregulated in BCSCs (Table 2), and previously we reported it as a potential chemoresponse regulator [39]. Literature shows that ITGA7 has features in breast of both a tumour suppressor, for example, reduced expression in cancer compared to normal tissue [37] and reduced expression in metastases [66], and an oncogene, for example high expression linked to poor survival [67] and knock-down in vitro associated with reduced proliferation or invasion [37, 67]. This confusion may again relate different roles within stem vs non-stem cancer cells, or within stromal cells vs cancer cells. We found relatively low ITGA7 expression specifically within cancer cells to be associated with poor patient outcomes after chemotherapy (Fig. 3), which was concordant with reduced expression indicating increased stem-like properties including chemoresistance. Furthermore, we confirmed this impact on chemoresistance using in vitro siRNA knock-downs (Fig. 4); in fact, our finding that ITGA7 knock-down led to relative chemoresistance is compatible with previous reports that knock-down led to reduced proliferation [37, 67], since lower proliferation is linked to both resistance to cytotoxics and stem cell phenotypes [9]. Interestingly, we found ITGA7 to be expressed in both the plasma membrane/cytoplasm and in the nucleus, as has been reported for a growing list of integrins [44–46], although not previously for ITGA7. The molecular function of nuclear ITGA7 remains unclear, but it should be noted that expression in this compartment significantly correlated with patient outcomes (Fig. 3e, f), therefore it is likely to be functional. The best characterised example of nuclear integrins is ITGAV/ITGB3 in ovarian cancer [45]; in this case, nuclear localisation was cancer-specific, and induced proliferation without interfering with the adherence function of the plasma-membrane located fraction. Importantly, we do not find that low ITGA7 alone is a viable marker of BCSCs, as is evident from the complete absence of expression in any cancer cells in many cancer cases (Fig. 3d), but that low ITGA7 is associated with some stem-like behaviours such as chemoresistance.

In summary, we have determined the first statistically significant transcriptome profile of stem-like cells from primary breast cancers. We expect this profile to guide future experimental assessment of novel markers and therapeutic targets, based on assessing or targeting BCSCs. Using the profile, we have identified ITGA7 as a mediator of the stem-like property of chemoresistance, and define ITGA7 as a predictive marker for chemoresponse in breast cancer, thereby highlighting integrins for future study in order to consider novel chemo-sensitisation strategies.

## Supplementary information

S material

Table S3

Table S4

Video S1

Reproducibility checklist

## Data Availability

All data are available either within the manuscript and Supplementary material, or directly from the corresponding author.
