# Supplementary material for: Transcriptome profiles of stem-like cells from primary breast cancers allow identification of ITGA7 as a predictive marker of chemotherapy response
Source: Br J Cancer. 2021 Jul 12;125(7):983–93. doi: 10.1038/s41416-021-01484-w (PMC8476506; doi:10.1038/s41416-021-01484-w)
Supplement: Supplementary file 1 — S material [file 41416_2021_1484_MOESM1_ESM.pdf]

Supplementary material:

- **Figures S1 – S8**
- **Video S1 legend**
- **Tables S1 – S7 (for S3 and S4, legends only)**
- **Supplementary methods**

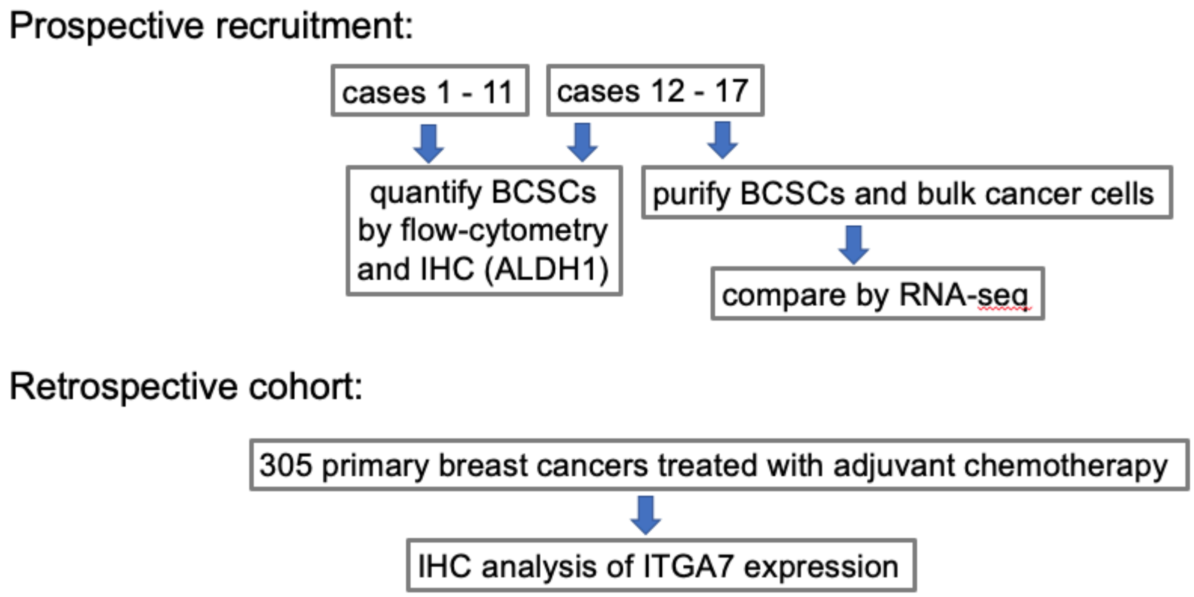

Fig S1. A flow-scheme depicting our recruitment of patients and use of patient samples. BCSCs, breast cancer stem-like cells; IHC, immunohistochemistry

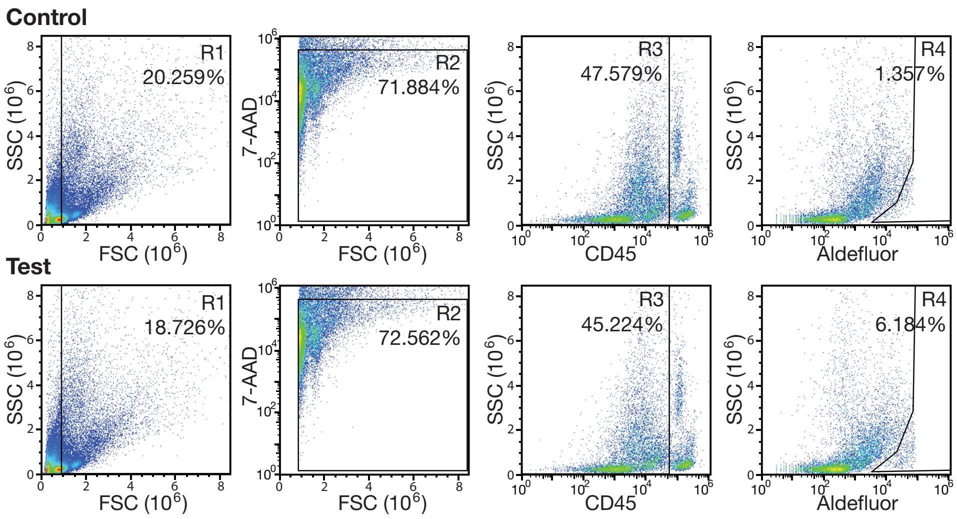

Figure S2. Representative flow-cytometry plots showing the gating strategy to define BCSCs (Aldefluor positive) and bulk (Aldefluor negative) cells. Single cell suspensions were prepared from fresh breast cancer core biopsies. Stem-like cells were labelled based on functional ALDH activity using the Aldefluor assay either without (test) or with (control) a specific inhibitor of ALDH, DEAB. Cells were also labelled with 7-Aminoactinomycin D (7-AAD) and fluorescently-tagged anti-human CD45. Cells were gated on live nucleate cells on the basis of forward scatter (FSC)/side-scatter (SSC) (R1), live cells by excluding those positive for 7-AAD (R2), and non-hemopoietic cells by excluding those positive for anti-CD45 (R3). BCSCs (Aldefluor positive cells) were defined using gates set for each individual sample, accepting ~1% positivity in the DEAB-inhibited control. % values shown are the proportion of cells selected within that plot. This example is case 6 (Table 1).

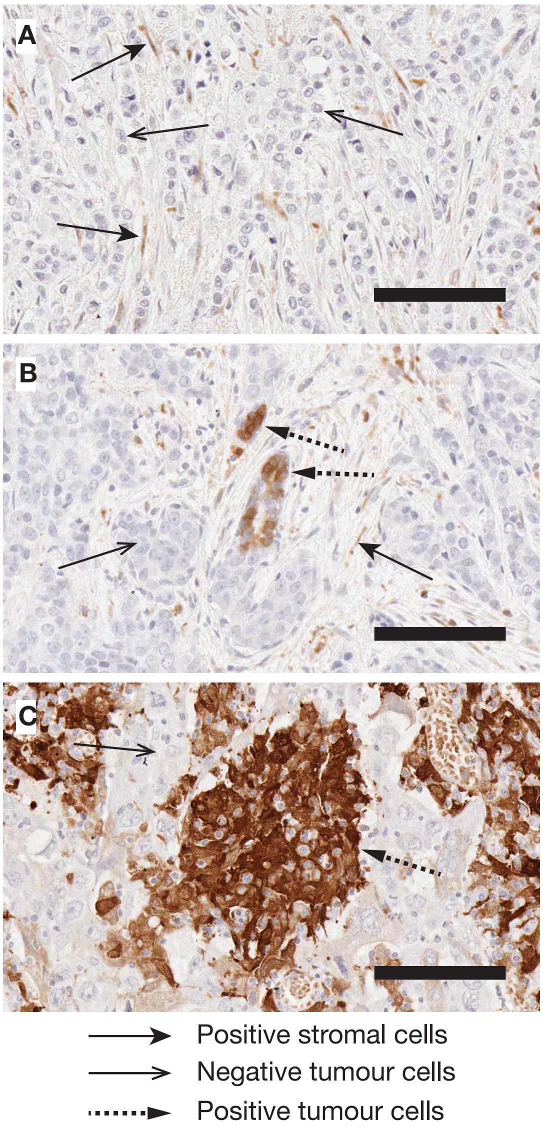

Figure S3. Representative immunohistochemistry images of ALDH1 staining. A) An invasive lobular carcinoma, classic variant (case 4; Table 1), showing negative staining of tumour cells for ALDH1 (staining scored as 0), with positive staining of stromal cells (arrows). B) An invasive ductal carcinoma, NST (case 10; Table 1), showing moderate positive cytoplasmic staining of 10% of tumour cells (dotted arrows showing positive cells, solid arrows with thin head showing negative cells; staining scored as 4). Stromal staining also visible (solid arrow with solid head). C) An invasive ductal carcinoma, NST (case 7; Table 1), showing strong positive cytoplasmic staining of 65% of tumour cells (dotted arrows showing positive cells, solid arrows with thin head showing negative cells; staining scored as 12). NST, No special type

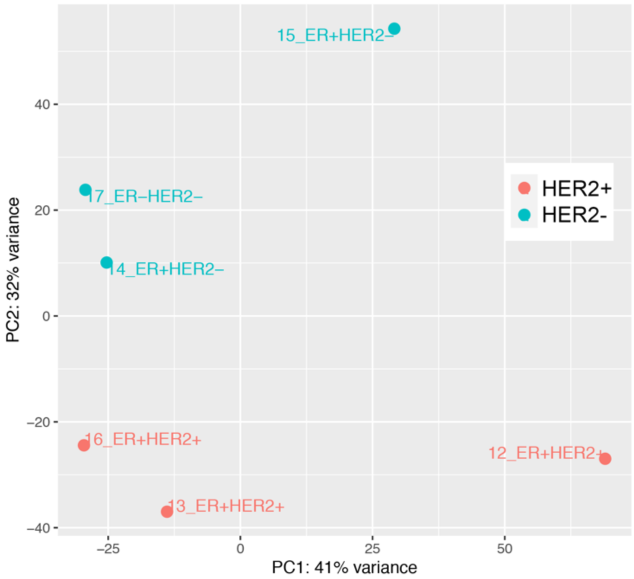

Figure S4. Principal component analysis of BCSC samples, noting receptor status. BCSCs were sorted by FACS from 6 cancers (cases 12 through to 17). Expression profiles were determined by RNA-seq. Principal component analysis of transcriptomes was performed to investigate the relationships between the samples. Datapoints representing each case are numbered (12-17), coloured according to HER2 status (red: HER2-positive; blue: HER2-negative), and labelled to show ER status (positive: +; negative: -).

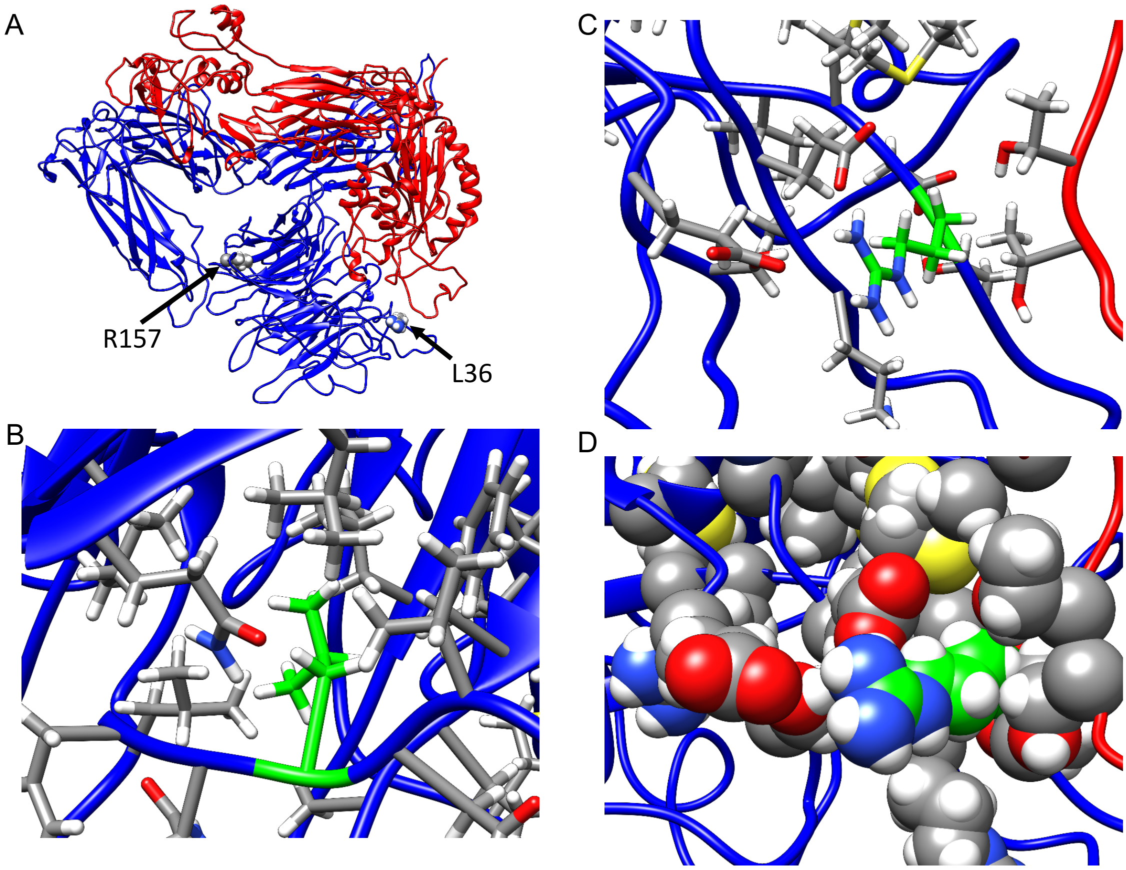

Figure S5. L36V and R157Q variants in ITGA7 occur in structurally important regions of the protein. A) Homology model of the ITGA7/ITGB1 complex, showing chain ITGA7 in blue and ITGB1 in red in secondary structure representation. The positions of L36 and R157 are indicated with space filling representation, and are coloured by element. B) Close up in sticks representation of L36 (sidechain heavy atoms shown in green) and neighbouring hydrophobic residues. The ITGA7 backbone is depicted in blue in secondary structure representation. C) Close up in sticks representation of R157 interaction with neighbouring negatively charged E120 and E145. ITGA7 and ITGB1 backbones are shown in secondary structure representation in blue and red respectively. D) Close up in space filling representation of R157 interaction with neighbouring negatively charged E120 and E145. ITGA7 and ITGB1 backbones are shown in secondary structure representation in blue and red respectively.

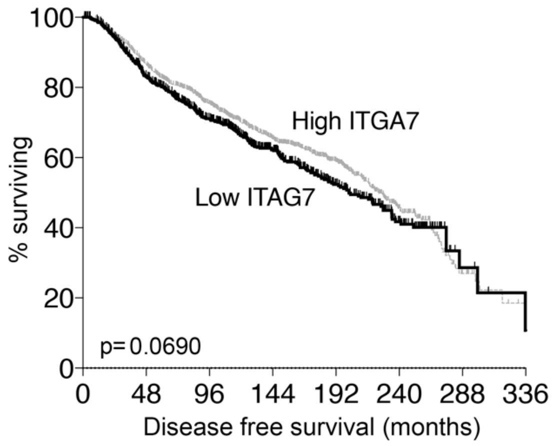

Figure S6. ITGA7 expression did not significantly correlate with disease free survival in breast cancer in a cohort treated with a variety of regimens. METABRIC transcriptomic data for breast cancers were accessed via cbioportal and records with ITGA7 expression data and suitable clinical annotation were identified (n=1903). Cases were dichotimised into low and high ITGA7 expression groups using receiver operator curve analyses. Kaplan-Meier survival analysis was performed; p values were determined using log rank tests.

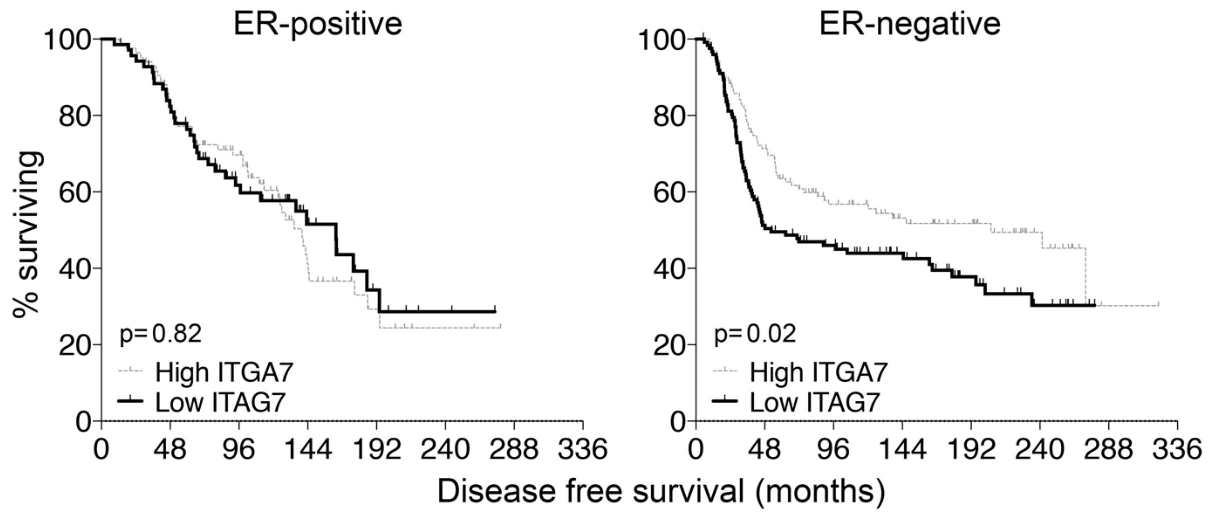

Figure S7. ITGA7 expression correlated significantly with disease free survival after chemotherapy in ER-negative but not ER-positive breast cancers. METABRIC transcriptomic data for breast cancers were accessed via cbioportal and records with ITGA7 expression data and suitable clinical annotation, and who received chemotherapy were identified (n=398). Cases were split into ER-positive (left plot; n=154) and ER-negative (right plot; n=244) and were dichotomised into low and high ITGA7 expression groups using receiver operator curve analyses. Kaplan-Meier survival analyses were performed; p values were determined using log rank tests.

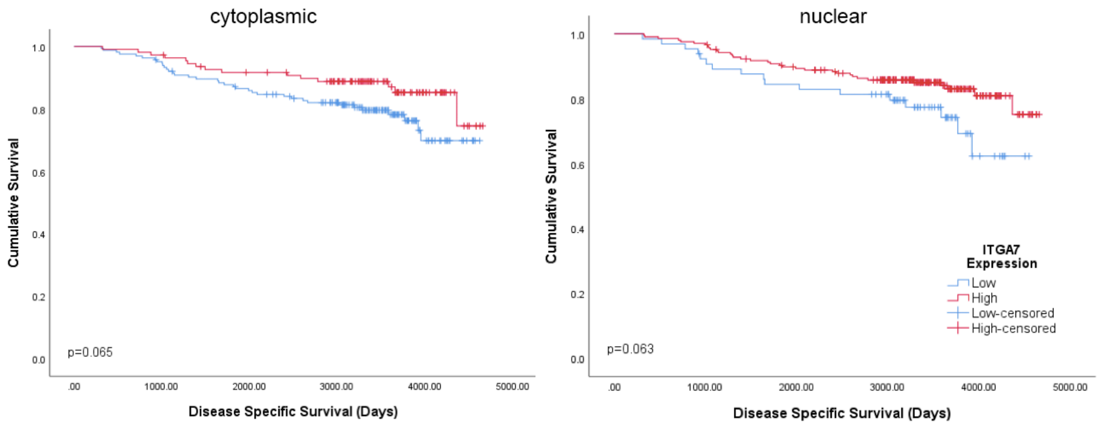

Figure S8. Low ITGA7 protein expression was not significantly correlated with disease specific survival after chemotherapy in either cytoplasmic or nuclear compartments. ITGA7 expression in cytoplasm and nucleus were scored separately in breast tumour resection tissue from 305 breast cancer patients subsequently treated with adjuvant chemotherapy. Cases were dichotomised into two groups based on low or high expression using cut offs defined by receiver operator curve analyses. Kaplan–Meier survival analyses were performed to determine whether expression of ITGA7 in either compartment was significantly related to disease specific survival; p values were determined using log rank tests.

Mp4 movie file

Video S1. L36V and R157Q variants in ITGA7 occur in structurally important regions of the protein. Animation showing the ITGA7/ITGB1 complex in secondary structure representation (coloured blue and red respectively). The two residues of interest (L36 and R157) are then shown in space filling representation, with the heavy atoms coloured green. The L36 residue is highlighted and the interactions with neighbouring hydrophobic residues are shown in space filling then stick representations. Next, the R157 residue is highlighted and the interactions with neighbouring hydrophobic residues are shown in space filling then stick representations. Finally, the complex is shown in a surface representation (ITGA7 in blue, ITGB1 in red).

CHARACTERISTICS	n=305 (100%)
Age: mean 52 (range 25-74) years	
Follow up: mean 120.9 (range 11-166.4) months	
**TUMOUR HISTOPATHOLOGY**	
Ductal NST	222 (72.8)
Lobular	23 (7.5)
Metaplastic	6 (2)
Other	6 (2)
Mixed	48 (15.7)
**TUMOUR GRADE**	
1	18 (5.9)
2	124 (40.7)
3	163 (53.4)
**LYMPH NODE STATUS**	
N0	117 (38.4)
N1	120 (39.3)
N2	42 (13.8)
N3	26 (8.5)
**RECEPTOR STATUS**	
ER+	207 (67.9)
HER2+	68 (22.3)
ER-, PR-, HER2- (triple negative)	68 (22.3)
**CHEMOTHERAPY REGIMENS**	
Anthracycline based	
- without Taxanes	149 (48.9)
- with Taxanes	116 (38)
- with others	40 (13.1)

Table S1. Summary of the clinico-pathological and pharmacological features for the cohort of breast cancer patients receiving adjuvant chemotherapy

Sample	Reads mapping to transcripts
P12 -	18,453,716
P12 +	22,173,284
P13 -	15,748,214
P13 +	32,821,810
P14 -	13,335,896
P14 +	24,511,048
P15 -	12,585,520
P15 +	7,649,654
P16 -	17,561,240
P16 +	26,961,590
P17 -	17,591,772
P17 +	14,819,392

Table S2. Sequencing metrics: numbers of RNA-seq reads aligning to transcripts. RNA-seq was performed on Aldefluor positive (+) and Aldefluor negative (-) tumour cells purified from 6 breast cancers (Patient, P12-17) using flow-cytometry. The numbers of sequencing reads successfully aligning to the RefSeq transcript dataset are shown.

Excel spreadsheet

Table S3. Transcripts that were significantly differentially-expressed in BCSCs vs matched bulk tumour cells. Expression profiles were compared in matched pairs of BCSCs and bulk tumour cells purified from primary human breast cancers by flow-cytometry. Analyses were performed on all 6 matched pairs or only 5 pairs (excluding P14), in separate tabs. The table identifies the gene name (SYMBOL), the transcript reference number (RefSeq), the mean log2-transformed fold change in expression (log2FoldChange; negative numbers indicate down-regulated in BCSCs), the p value representing significance of differential expression as either corrected for multiple testing (padj) or uncorrected (pvalue).

Excel spreadsheet

Table S4. Gene ontology terms that were significantly enriched in the genes differentially-expressed between BCSCs vs matched bulk tumour cells. Genes that were differentially-expressed between BCSCs and matched bulk tumour cells were identified; analyses were performed on all 6 matched pairs or only 5 pairs – see Table S2. Gene lists were then tested for significant over-representation of associations with gene ontology terms, in comparison to the pooled transcriptome from all samples. The table identifies the gene ontology reference (GOBPID), the p value associated with the significant over-representation (Pvalue), the number of genes from that ontology group that would be expected by chance in a random input of the same size (ExpCount), the actual number of genes within the input matching the ontology group (Count), the total number of genes curated as associated with the ontology group and expressed within the pooled transcriptome (size), and the term description (Term).

		Tumour grade (1, 2 or 3)	Lymph node status (positive/negative)	Estrogen receptor status (positive/negative)
**Cytoplasmic ITGA7**	**r**	-0.104	-0.01	0.114
	**p**	0.086	0.989	0.059
**Nuclear ITGA7**	**r**	-0.122	0.004	0.045
	**p**	0.044*	0.944	0.456

Table S5. ITGA7 nuclear expression correlates with tumour grade in whole cohort. Spearman's rho analyses were performed for ITGA7 expression levels in the cytoplasmic or nuclear compartments against the prognostic factors shown. Spearman's rho coefficients (r) and p values (p) are shown. * denotes a significant correlation as a single text, although significance is lost when this is corrected multiple testing (6 tests in the panel).

	ROC defined cut offs
**ITGA7 cytoplasm**	6.5
**ITGA7 nucleus**	2.5

Table S6. Cut off values defined by receiver operator curve analyses to dichotomise cohorts into relatively low and relatively high groups.

Expression		Mean DFS (days)	Log rank	Mean DSS (days)	Log rank
**Cytoplasmic ITGA7**	**Low**	3431	0.64	3581	0.87
	**High**	3432		3499	
**Nuclear ITGA7**	**Low**	3191	0.51	3701	0.64
	**High**	3564		3560	

Table S7. ITGA7 expression does not correlate with outcomes in ER negative breast cancers. Mean disease-free survival (DFS) and disease specific survival (DSS) values are shown for groups with low or high expression of ITGA7 in ER negative breast cancers (n=98). Survival was compared using log rank tests: p values are shown.

Supplementary methods

Preparation of single-cell suspensions from primary tumour samples

Single-cell suspensions were prepared using the Gentle MACS Dissociator (MACS Miltenyi Biotech, Bergisch-Gladbach, Germany). The enzymatic digestion was done using the Tumour dissociation kit, human (MACS Miltenyi Biotech, Bergisch-Gladbach, Germany). Briefly, an enzyme mix formed of 4.7ml RPMI 1640, 200 µl enzyme H, 100 µl enzyme R, 25 µl enzyme A, was placed into a MACS-C tube containing the tissue core biopsy. The tumour was cut into very small pieces (2-4 mm) with scissors inside the C-tube. The MACS-C tube was then attached to the MACS dissociator and run using the program h_tumour_01 followed by rotation using the tube rotator at 37ºC for 30 min.

Transcriptome profiling and analysis

Total RNA was extracted from BCSC (Aldefluor positive) or bulk cell (Aldefluor negative) populations using AllPrep DNA/RNA Mini kits (Qiagen; Hilden, Germany) following the manufacturer's instructions using a mean of 20,430 cells. RNA was analyzed for concentration/purity using the NanoDrop ND-1000 Spectrophotometer (Nanodrop technologies; Wilmington, USA), and for concentration/integrity using high sensitivity RNA Tape Station (Agilent; Santa Clara, USA). 100ng total RNA was used from each sample to produce a TruSeq stranded RNA Illumina compatible library from which rRNA was removed using rRNA-specific depletion reagents. Following size selection and adaptor removal with Ampbeads, library concentrations were determined by qPCR before combining to make an equimolar pool that was sequenced (150bp paired-end HiSeq3000 lane; Agilent; Santa Clara, USA). Sequence data were checked for lower quality bases and adaptor sequences with FastQC (https://www.bioinformatics.babraham.ac.uk/projects/fastqc/) before and after quality trimming using CutAdapt[1]. Trimmed data were then aligned against the hg38 human reference sequence using STAR[2]. Possible PCR duplicates were identified using Picard before alignment quality was determined using Qualimap[3]. Read counts for each transcript in RefSeq were performed using Rsubread[4]. Transcripts with >2 counts in >2 samples were retained, while rRNA transcript data were discounted. The resultant counts matrix was analysed using DeSeq2[5] to identify differentially expressed transcripts between Aldefluor positive and negative samples, using Wald tests with the Bonferroni correction for multiple testing. To account for inter-patient variation, each patient's samples were paired as a second parameter in DeSeq2. Gene symbols for the differentially expressed transcripts with a multiple testing adjusted $p<0.05$ were used to generate lists of gene names, which were then analysed by the R package GOstats[6] to identify over-represented gene ontology terms.

Immunohistochemistry

4μm (prospective cohort) or 5μm (retrospective TMAs) sections were taken onto SuperFrost plus slides (Menzel-Glaser; Braunschweig, Germany). Sections were dewaxed (xylene) and rehydrated (descending ethanol grades). Antigens were retrieved in 10mM citric buffer (pH 6) heated by microwave (full power, 10min). Slides were blocked in 0.3% hydrogen peroxide (Thermo Fisher; Waltham, USA) (10min), and washed in Tris-Buffered Saline (TBS) and Antibody Diluent (Thermo Fisher; Waltham, USA). Slides were incubated with 1:50 mouse monoclonal anti-ALDH1 antibody (BD Biosciences; San Jose, USA) in Antibody Diluent (1h room temperature) or 1:100 rabbit polyclonal anti-ITGA7 antibody (ab75224; Abcam; Cambridge, USA) in Antibody Diluent (overnight at 4°C). Negative controls (lacking primary antibody) were performed. Slides were washed x3 in TBS. For ALDH1, IHC was completed using anti-mouse Envision reagents (Dako; Gostrup, Denmark) following the manufacturer's protocols, while for ITGA7 SignalStain Boost IHC detection Reagent (HRP, Rabbit) and SignalStain DAB substrate were used (Cell Signalling Technology; Massachusetts, USA). Slides were counterstained with Mayer's Haematoxylin (2min). Finally, slides were washed in Scott's water (1min) before dehydration (ascending grades of ethanol) and clearing (xylene). DPX (Fluka; Gillingham, UK) was used for mounting. Sections were digitally scanned using ScanScopeXT (20x) and scored using Webscope (Aperio; Vista, USA). NG (specialist histopathologist) scored ALDH1. Cytoplasmic ALDH1 expression in tumour cells was assessed in terms of percentage of positively stained tumour cells (P: 0% score 0; >0%-1% score 1; >1%-10% score 2; >10%-33% score 3; >33-66% score 4; >66% score 5) and staining intensity (I: no staining 0; weak 1; moderate 2; strong 3). Quick score was used, as previously for ALDH1[7,8], by multiplying P and I, giving totals of 0-15. ALDH1 staining of stromal cells was fairly frequent and was considered a positive internal control[7]. For ITGA7, SJJ and RAM-S (breast consultant histopathologist) scored and the scoring protocol was developed in consultation with RAM-S. Cytoplasmic and nuclear staining were scored separately, based on intensity and proportion using scoring thresholds as for ALDH1. In this case, intensity and proportion scores were added giving final scores of 0-8. SJJ score all cores, while RAM-S scored 10% of the tumour cores independently; Cohen's Kappa statistic was used to determine the inter-scorer concordance – this indicated near perfect agreement (0.83 for nuclear and 0.88 for cytoplasmic ITGA7), demonstrating that scoring was robust and reproducible. ITGA7 scores for each case were means of scores from each core representing that case.

Methods references

1 Martin, M. Cutadapt removes adapter sequences from high-throughput sequencing reads. *EMBnet.journal* **17**, 10-12 (2011).

2 Dobin, A., Davis, C. A., Schlesinger, F., Drenkow, J., Zaleski, C., Jha, S. *et al.* STAR: ultrafast universal RNA-seq aligner. *Bioinformatics* **29**, 15-21 (2013).

3 Okonechnikov, K., Conesa, A. & Garcia-Alcalde, F. Qualimap 2: advanced multi-sample quality control for high-throughput sequencing data. *Bioinformatics* **32**, 292-294 (2016).

4 Liao, Y., Smyth, G. K. & Shi, W. The R package Rsubread is easier, faster, cheaper and better for alignment and quantification of RNA sequencing reads. *Nuc. Acids Res.* **47**, e47 (2019).

5 Love, M. I., Huber, W. & Anders, S. Moderated estimation of fold change and dispersion for RNA-seq data with DESeq2. *Genome biology* **15**, 550 (2014).

6 Falcon, S. & Gentleman, R. Using GOstats to test gene lists for GO term association. *Bioinformatics* **23**, 257-258 (2007).

7 Charafe-Jauffret, E., Ginestier, C., Iovino, F., Tarpin, C., Diebel, M., Esterni, B. *et al.* Aldehyde dehydrogenase 1-positive cancer stem cells mediate metastasis and poor clinical outcome in inflammatory breast cancer. *Clin Cancer Res* **16**, 45-55 (2010).

8 Ginestier, C., Hur, M. H., Charafe-Jauffret, E., Monville, F., Dutcher, J., Brown, M. *et al.* ALDH1 is a marker of normal and malignant human mammary stem cells and a predictor of poor clinical outcome. *Cell Stem Cell* **1**, 555-567 (2007).
